# Supplementary material for: A Structurally Characterized Staphylococcus aureus Evolutionary Escape Route from Treatment with the Antibiotic Linezolid
Source: Microbiol Spectr. 2022 Jun 23;10(4):e00583-22. doi: 10.1128/spectrum.00583-22 (PMC9431193; doi:10.1128/spectrum.00583-22)
Supplement: Supplemental file 1 — Supplemental material. Download spectrum.00583-22-s0001.pdf, PDF file, 1.0 MB [file spectrum.00583-22-s0001.pdf]

SUPPLEMENT

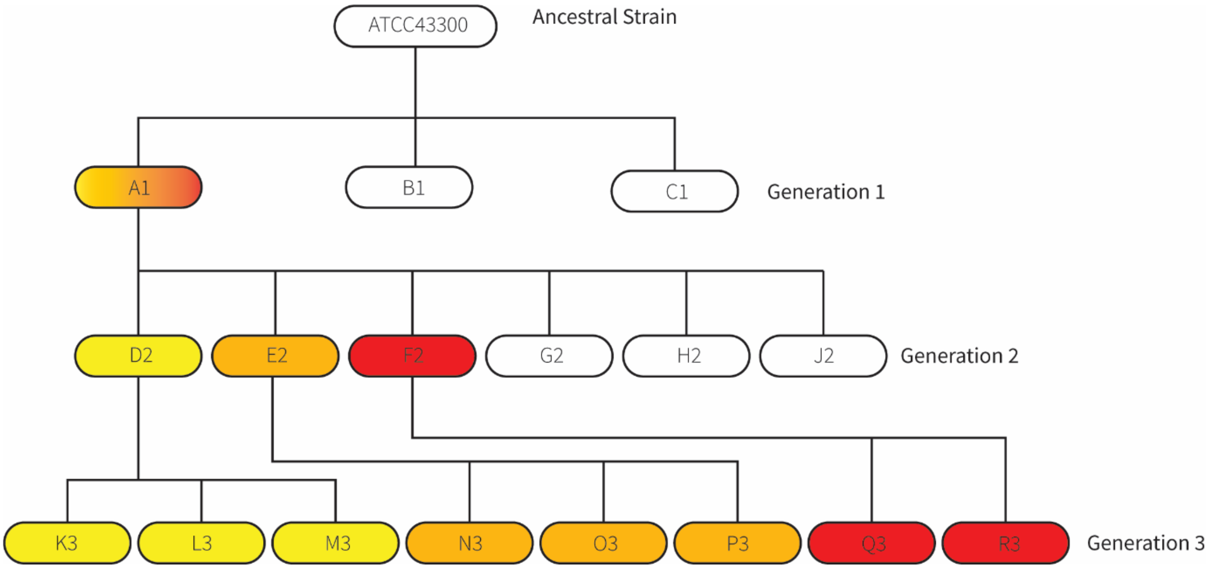

Supplemental Figure S1. Graphical representation of strain lineage in the antibiotic selection experiment.
The three generations of the experiment are designated 1, 2 and 3. Each of the strains in the lineages were labelled alphabetically. The colors denote the strain lineages: yellow showing strains derived from D2, orange showing strains derived from E2 and red showing strains derived from F2.

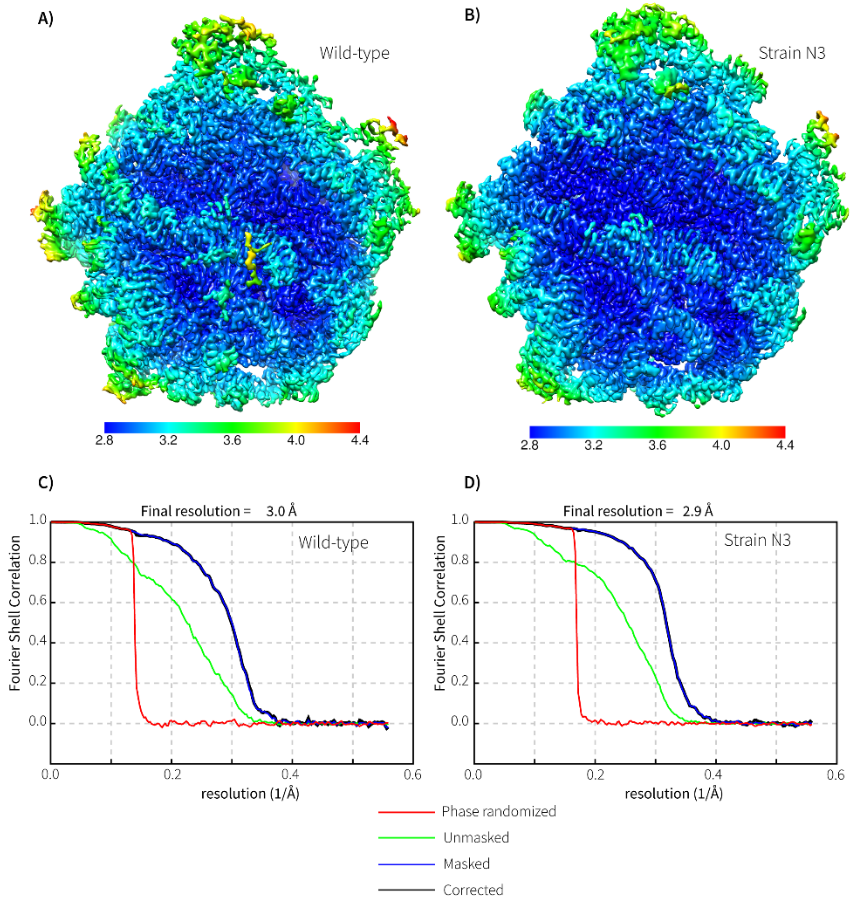

Supplemental Figure S2. CryoEM validation and map quality.
A) Local Resolution map of the 50S ribosomal subunit for the Wild-type *S. aureus* (ATCC43300). **B)** Local Resolution map of the 50S ribosomal subunit for the Strain N3 *S. aureus*. **C)** Fourier Shell Correlation (FSC) plots for the Wild-type 50S focussed map. **D)** FSC plots for the Strain N3 50S focussed map.

Supplemental Table S1. Calculated growth rates of *S. aureus* strains on three growth media. The rate, k_{obs}, was calculated using the PRISM software package. Rate is reported in $hours^{-1}$.

Strain	***ATCC43300***	***Strain A1***	***Strain D2***	***Stain E2***	***Strain L3***	***Strain N3***	***Strain R3***
LB	0.84	0.57	0.66	0.67	0.66	0.23	0.66
BHI	0.77	0.76	0.74	0.70	0.59	0.42	0.64
MHB	0.70	0.45	0.60	0.56	0.39	0.15	0.56

Supplemental Table S2. Single nucleotide variants (SNVs) for each genome. **Chrom**: contig name of the reference genome. **Pos:** position of the variant in the reference genome. **Ref:** Nucleotide in the reference genome. **Alt:** Nucleotide Variant. **SNV_effect**: effect of the variant in the codon translation. **GeneReferenceID**: Gene ID. **GeneName:** Gene name. **ntChange:** nucleotide change**. aaChange:** aminoacid change caused by the nucleotide variant. The values 0,1 represent Ref and Alt, respectively.
